# Advancing Regional and Remote Health Care With Virtual Hospital Implementation: Rapid Review

**DOI:** 10.2196/64582

**Published:** 2025-06-03

**Authors:** Artika Archana Kumari, Tafheem Ahmad Wani, Michael Liem, James Boyd, Urooj Raza Khan

**Affiliations:** 1 Department of Public Health School of Psychology and Public Health La Trobe University Bundoora Australia

**Keywords:** virtual hospitals, regional and remote health care, health care accessibility, patient experiences, health care provider perspectives, rural health care, telehealth, virtual care from home, telemedicine, health system outcomes, clinical outcomes, barriers and facilitators, recommendations

## Abstract

**Background:**

Disparities in health equity between metropolitan and rural areas are a global concern, especially in vast countries such as Australia, Canada, and the United States. Virtual care models in health care settings are promising in reducing inequalities, with virtual hospitals (VHs) potentially bridging the gap for isolated or underserved regions. However, evidence-based strategies and the complexities of VH implementation necessitate further research.

**Objective:**

This rapid review aims to examine the role of VHs in enhancing regional and remote health care by focusing on accessibility, patient and health care provider experiences, and implementation barriers and facilitators. It provides tailored recommendations for large-scale implementation in communities with access issues, contributing to the discussion on equitable health care.

**Methods:**

A rapid review was conducted in accordance with the World Health Organization guidelines. A systematic search was performed across PubMed, MEDLINE, CINAHL, and the La Trobe University Library for peer-reviewed articles published between January 2015 and March 2023. Additional gray literature was identified through Google searches and snowballing from relevant web articles. Studies were included if they focused on regional or remote populations and addressed VHs or virtual care. Studies that solely discussed hybrid models of care were excluded. Data were systematically extracted using a customized Microsoft Excel template. A mixed methods thematic analysis was conducted to identify recurring themes, barriers, facilitators, and recommendations related to VH implementation as well as patterns in clinical outcomes and stakeholder perspectives.

**Results:**

A total of 35 articles were included in this review, comprising 23 (66%) peer-reviewed studies and 12 (34%) gray literature sources. Positive clinical outcomes were reported in 9 (26%) articles, highlighting outcomes such as reduced disease transmission, improved patient safety, fewer admissions and readmissions, lower mortality, shorter hospital stays, and better adherence to clinical best practices. Health system outcomes were identified in 15 (43%) articles, including reduced costs, enhanced patient experience and safety, improved care delivery and health care provider support, greater efficiency, broader geographic coverage, and better integration of services. Patient and health care provider perspectives were discussed in 12 (34%) articles, with positive views attributed to convenience, time and cost savings, and improved service quality. Barriers and facilitators were the most frequently discussed themes, appearing in 27 (77%) and 26 (74%) articles, respectively, with challenges and enablers commonly linked to people, processes, technology, and financial sustainability.

**Conclusions:**

VHs have the potential to revolutionize regional and remote health care by overcoming barriers, using facilitators, and following recommended practices, leading to better clinical outcomes and increased satisfaction for patients and health care providers.

## Introduction

### Background

Health care disparities between rural and remote regions and urban centers are a global challenge. Countries such as Australia, Canada, and the United States have documented significant disparities in health care access and outcomes [1-3]. Data from the Australian Institute of Health and Welfare reveal a 1.4-fold increased incidence of disease in remote and very remote regions compared to urban cities [2]. Furthermore, in very remote regions, the risk of premature mortality is elevated by 1.3 times for men and 1.5 times for women compared to metropolitan areas. This disparity aligns with findings from the Centers for Disease Control and Prevention, which highlights the challenges faced by rural Americans due to limited access to specialized and emergency health care, ultimately contributing to a higher prevalence of preventable deaths in these communities [4].

Traditional health care models often struggle to provide equitable services in geographically isolated regions due to shortages of medical facilities, specialized professionals, and advanced diagnostic equipment. Consequently, rural and remote populations experience higher rates of preventable diseases, avoidable hospitalizations, and mortality compared to urban counterparts [2,4,5]. In Australia, hospitalization rates for preventable conditions are 2.5 times higher [2]. Similarly, in the United States, rural populations have a 20% higher overall mortality rate compared to urban counterparts, and 1 in 5 rural adults experience multiple chronic conditions, leading to increased rates of preventable hospitalizations and deaths [5].

**Figure 1:**
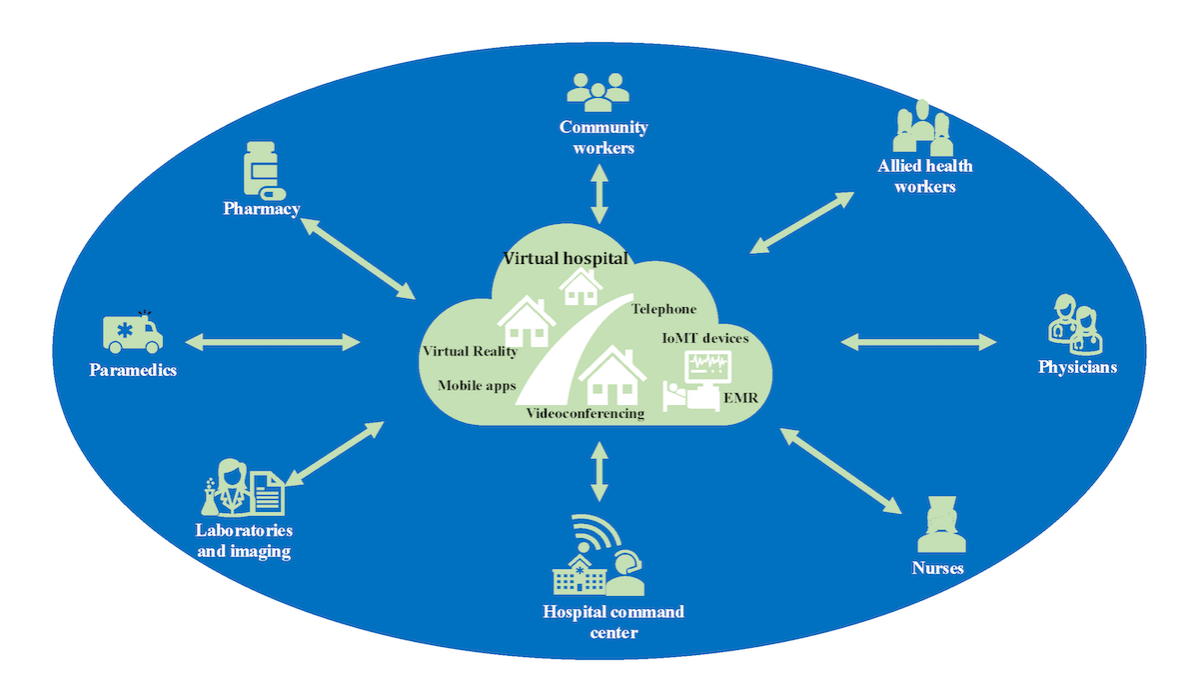
Concept of a virtual hospital. IoMT: Internet of Medical Things; EMR: electronic medical record.

The emergence of virtual health offers a promising solution to bridge the health care gap in regional and remote locations. Virtual health, driven by digital technologies, has made considerable strides, transforming primary, secondary, and tertiary care services. Health care institutions have embraced virtual health solutions, such as virtual hospitals (VHs), to provide advanced treatments, surgical consultations, multidisciplinary team discussions, and hospital-at-home care, enhancing patient access to complex medical interventions. VHs leverage digital initiatives, such as telehealth and telemedicine technologies, to deliver a comprehensive range of remote health care services, including consultations, diagnostics, treatment plans, and hospital-level care at home [6]. These services are typically delivered through videoconferencing, web platforms, mobile apps, and other digital channels. Figure 1 further illustrates the concept of a VH.

Unlike traditional hospitals with physical infrastructure, VHs operate primarily in the digital realm. Their significance lies in their ability to transcend geographic barriers, enhancing access to health care for patients in remote or underserved areas [7]. It is important to note that while the terms VH, virtual care, hospital at home (virtual wards), and telehealth are sometimes used interchangeably, there are key distinctions between them. Virtual care represents a broad spectrum of health care services delivered remotely, encompassing a range of synchronous and asynchronous technologies and applications. Talevski et al [8] states that virtual care refers to “the integration of digital technologies across the continuum of care to improve patient outcomes.” This definition covers existing health care programs, such as the Victorian Virtual Emergency Department [8], the Ochsner Connected Health Remote Patient Monitoring (RPM) Program [9], and the Cleveland Clinic Express Care Online [10]. Telehealth, on the other hand, is a subset of virtual care that primarily focuses on real-time communication between patients and providers using telecommunication technology [11]. An example of virtual care outside the scope of telehealth is RPM. RPM systems use devices, such as wearables or sensors, to collect and transmit health data, such as heart rate or glucose levels, to health care providers for analysis [12]. This occurs asynchronously, making it distinct from the real-time communication that characterizes telehealth. On the other hand, VH represents a more comprehensive approach to remote health care, offering a wide range of services beyond consultations, including remote diagnostics, treatment plan development, and virtual wards [13]. Hospital-at-home programs, a subset of VH or stand-alone initiatives, provide hospital-level care in the patient’s home, incorporating remote monitoring and regular consultations [14].

Digital health technologies form the cornerstone of modern health care delivery systems, including virtual care, telehealth, VHs, and hospital-at-home programs, facilitating remote health care services through diverse technological platforms and applications. According to the World Health Organization [15], digital health encompasses “the field of knowledge and practice associated with the development and use of digital technologies to improve health”. This definition encompasses the various terms often used when discussing the design and implementation of technology in health care, including virtual care, telehealth, and VHs. The Healthcare Information and Management Systems Society [12] further characterizes digital health as a comprehensive framework that incorporates various digital technologies, systems, and platforms designed to deliver health care, manage health information, and enhance patient care delivery.

The COVID-19 pandemic accelerated the adoption of VHs as a means to manage health care demands while mitigating infection risks. This rapid expansion led to demonstrated improvements in patient outcomes, including length of stay (LOS) and improved wait times [16-18]. Success stories, such as the Royal Prince Alfred VH [19], the Mayo Clinic Advanced Care at Home program [20], and the Victorian Virtual Emergency Department [21], further exemplify the capability of VHs to deliver effective health care remotely. The experience gained during the pandemic highlights the need for strategic planning and investment in VHs to address health care disparities in rural and remote areas [22,23].

The successful implementation of a VH is a complex endeavor, requiring careful planning, assessment, and integration of digital technologies [24]. Challenges such as inadequate guidance, poor governance, limited technological literacy, and concerns about safety and privacy can hinder the seamless adoption of VHs and related virtual care-from-home solutions [17,18]. To overcome these barriers and successfully implement VHs, evidence-based recommendations must be prioritized [25].

### Research Focus and Aims

Despite the potential of VHs to transform health care delivery, there is a notable lack of evidence supporting context-specific and comprehensive implementation strategies of these systems [26]. Existing research often focuses on individual virtual care programs rather than the broader array of integrated health care technologies used in VHs [27]. To effectively deliver VH services, a deeper understanding of how different virtual care technologies contribute to overall patient care is essential. This knowledge is critical for optimizing the design, implementation, and scalability of VH services, particularly in rural and remote regions.

Current evidence highlights the potential of VHs to revolutionize health care delivery by offering improved clinical and health system outcomes, enhanced provider and patient experiences, and increased health care accessibility. However, this evidence is largely limited to the broader health care landscape, with a noticeable gap in understanding the specific application of VHs in regional and rural settings. Existing studies discuss the clinical effectiveness of VHs but primarily address the general population [28,29]. Similarly, research on the barriers, facilitators, and recommendations for implementing VHs does not align with the unique challenges of regional and remote communities, such as limited access to specialists, longer travel distances, and a higher prevalence of preventable conditions [17,18]. This gap in the literature necessitates research focused on tailored recommendations, barriers, and facilitators specific to VH implementation in regional and remote areas, enabling health care systems to fully harness the potential of this innovative approach.

This rapid review aims to synthesize existing evidence on the role of VHs in enhancing health care delivery in regional and remote areas. It seeks to analyze clinical and health system outcomes, explore patient and provider perspectives, identify implementation-related barriers and facilitators, and provide recommendations for its successful implementation. The findings will contribute to evidence-based policy making and decision-making to optimize the use of VHs in addressing health care disparities in challenging geographic settings.

## Methods

### Overview

This rapid review was conducted to assess the potential of VHs in addressing health care disparities in regional and remote areas. This approach aligns with the need to understand how VHs address health care disparities and improve access for remote and underserved populations in a timely manner. By analyzing a wide range of literature, a rapid review helped to identify the most current evidence and trends, providing a comprehensive overview of the benefits, challenges, and best practices associated with developing VHs to support regional and remote health care.

Using the World Health Organization rapid review guideline [30] and the 2020 PRISMA (Preferred Reporting Items for Systematic Reviews and Meta-Analyses) statement [31], a systematically defined, transparent, and concise approach was used to identify relevant peer-reviewed articles and gray literature, extract relevant information, and synthesize the findings of this review. A completed PRISMA checklist is provided in Multimedia Appendix 1.

### Protocol Development

The research protocol for this study was developed using the population, intervention, comparison, and outcome (PICO) framework [32]. The protocol, as presented in Table 1, guided the development of the search strategy (Multimedia Appendix 2). La Trobe librarians also contributed to the development of the protocol and search strategy.

**Table 1:** Population, intervention, comparison, and outcome protocol development.

Parameter	Description	Keywords
Population	Patients living in regional and remote areas Patients having limited access to health care due to distance and a lack of resources	Regional health, rural health, remote health, rural health services, and remote and regional area health
Intervention	Building VH^a^ using telemedicine, telehealth, or eHealth technologies	Virtual hospital, telemedicine, telehealth, telemonitoring, remote consultation, virtual care, virtual health, eHealth, mHealth, and health virtualization
Comparison	Physical public and private hospitals (traditional health care delivery models) without VHs or digital health technologies	—^b^
Outcome	Improved health care access, quality of care, patient satisfaction, health care provider satisfaction, and health outcomes and reduced health care costs	Health care delivery, health care access, health planning, hospital strategy, health care accessibility, clinical effectiveness, user experience, implementation, best practices, recommendations, enablers, strategies, benefits, facilitators, challenges, barriers, problems

^a^VH: virtual hospital.

^b^Not applicable.

### Search Strategy

We searched for peer-reviewed articles from January 2015 to March 2023 across 3 key databases: PubMed, MEDLINE, and CINAHL. The La Trobe University Library database was also used. The time frame from January 2015 to March 2023 was intentionally chosen to capture the literature spanning both the prepandemic development of VH models and the significant innovations that emerged in response to the COVID-19 pandemic.

Our search strategy was designed to align with the PICO framework, using 2 distinct approaches. The first strategy targeted articles discussing enablers and facilitators of VH implementation, while the second strategy focused on identifying literature addressing barriers, challenges, and limitations. In addition, to ensure quality and minimize bias, both search techniques were reviewed collaboratively by all authors in consultation with La Trobe research librarians.

To identify relevant gray literature, the research topic “advancing regional and remote health care with virtual hospital implementation” was googled, and from the first 4 pages of the search result, policy documents and web articles that discussed the research questions were marked as relevant and selected for further analysis. Furthermore, snowballing was used to screen and select other gray literature cited in relevant web articles [33].

### Inclusion and Exclusion Criteria

A set of inclusion and exclusion criteria was developed using the PICO framework (Textbox 1). This review included (1) studies addressing regional or remote populations or populations facing health care access difficulties; (2) studies discussing recommendations, strategies, facilitators, barriers, challenges, or limitations to VH or virtual care-from-home solutions, telehealth, telemedicine, eHealth, mobile health, and remote monitoring for hospital-level care at home; (3) studies discussing clinical or health system outcomes, user experience, and patient perspectives of the innovative technologies used in developing VHs and (4) studies conducted in a country such as Australia or countries with similar geographic landmasses, such as Canada, the United States, or India. Furthermore, only peer-reviewed studies published in English between January 2015 and March 2023 were included.

This review excluded (1) studies addressing urban or suburban population settings with comprehensive and timely health care access and (2) studies solely discussing hybrid models of care.

**Inclusion and exclusion criteria.**

**Inclusion criteria**

- Population: people of all age groups living in regional or remote regions and people with acute or chronic illness experiencing difficulties in accessing health care due to distance and lack of resources
- Intervention: virtual hospital (VH) or virtual care-from-home implementation; telemonitoring, telehealth, and telemedicine implementation supporting care-from-home services; eHealth or mobile health implementation; remote monitoring; interventions in a country such as Australia or countries with similar health care systems and geographic challenges; studies that discussed recommendations, opportunities, and challenges of implementing health care virtualization; and studies that discussed patient or provider experience or successful VH or virtual care-from-home implementation stories
- Comparison: an optional comparison group including people with no limitations to accessing health care but who preferred virtual consultation
- Outcomes: clinical outcomes of health care virtualization (improvements in health for people living in remote or regional areas or having limited access to resources); improved delivery of services; success strategies (stories) of VH implementation; program user experience (access, engagement, usability, experience, and satisfaction); and changes in health care resourcing
- Studies: published in peer-reviewed journals, conference proceedings, or health care services reports; published in the English language; and published between January 2015 and March 2023

**Exclusion criteria**

- Population: people living in urban or suburban areas and those having no limitation on health care access
- Intervention: traditional health care delivery models with minimum virtualization and virtualization in urban and suburban geographic regions
- Outcomes: studies discussing only the benefits and challenges of telehealth or telemedicine and studies discussing user perspectives only
- Studies: studies or reports from unverified sources that have not been peer reviewed, published in other languages, and published before January 2015 or after March 2023

### Screening and Study Selection

The search results were initially filtered for peer-reviewed articles published in English between January 2015 and March 2023. Following the initial database search, 589 records were retrieved from 4 databases. These records were imported into Covidence Systematic Review Software (Veritas Health Innovation) [34], where 255 (43.29%) duplicate records were eliminated automatically. The remaining 334 (56.71%) articles underwent title and abstract screening based on predefined inclusion and exclusion criteria. This stage resulted in the exclusion of 159 (47.6%) articles. The initial screening was conducted by AAK, with URK verifying a subset of the records to ensure consistency and accuracy in applying the criteria.

The remaining 175 full-text articles were then assessed for eligibility using the same inclusion and exclusion criteria. During this phase, the snowballing technique was also used, where relevant peer-reviewed articles cited within these studies were identified and retrieved from Google Scholar for potential inclusion. After full-text screening and the additional retrieval through snowballing, 23 (13.1%) articles met all eligibility criteria and were included in the final analysis.

### Data Extraction and Knowledge Synthesis

We used the Quality Criteria Checklist (Multimedia Appendix 3 [35]) to develop a Microsoft Excel template for quality assessment because our review included various study designs. This template helped us critically appraise the included studies and assess their potential bias.

In addition, a data extraction template was customized in Microsoft Excel (Multimedia Appendix 4), which was then used to systematically extract and record relevant information from the peer-reviewed studies and gray literature included. The template was prepared based on the extraction variables retrieved from the research question and objectives. Apart from the core characteristics, such as title, abstract, and author, the data extraction template included demographic location, health care conditions addressed, health care services provided virtually, virtual care technology used, health care outcomes, patient satisfaction, provider perspectives, barriers and facilitators, and recommendations and strategies for implementation. This approach helped ensure consistency and accuracy during the data extraction process. In addition, a concept matrix (Multimedia Appendix 5) was constructed to assess the relevance of each study to specific research objectives.

The extracted information from the included studies was then thematically analyzed to identify recurring patterns, emerging trends, and insights into common recommendations for developing VHs and their related barriers and facilitators. Data were also analyzed to identify common themes emerging from clinical and health care system outcomes and patient and provider perspectives. The thematic analysis approach allowed the identification and interpretation of key themes, enabling the synthesis and presentation of findings in a coherent and meaningful manner [36].

## Results

### Study Characteristics

**Figure 2:**
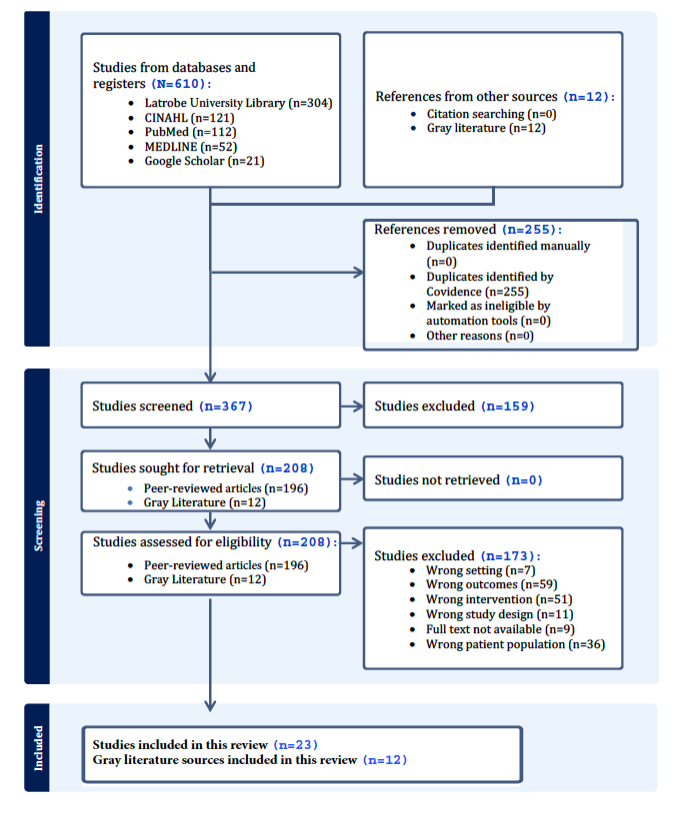
PRISMA (Preferred Reporting Items for Systematic Reviews and Meta-Analyses) diagram.

Overall, 23 peer-reviewed articles and 12 gray literature sources were included in our review. The selection process is summarized in the PRISMA flow diagram (Figure 2). Detailed characteristics of all included peer-reviewed articles and gray literature are presented in Tables 2 and 3. Among the 23 peer-reviewed articles, 12 (52%) focused on telehealth interventions [37-48], 6 (26%) discussed VHs or hospital-at-home interventions [49-54], 2 (9%) examined telemedicine [55,56], 2 (9%) addressed RPM [57,58], and 1 (4%) evaluated a virtual pharmacy [59]. In the 12 gray literature sources, 8 (67%) discussed virtual care-from-home solutions comprehensively [60-68], while 4 (33%) articles specifically addressed the concept of a VH [62,69-71].

**Table 2:** Included peer-reviewed articles.

Authors	Title	Country	Study design	Population	Health care conditions addressed	Intervention discussed
AlDossary et al [55], 2017	The development of a telemedicine planning framework based on needs assessment	United States	Case study	General rural population	Equitable access to specialty health care services	Telemedicine
Davis et al [38], 2020	Designing a multifaceted telehealth intervention for a rural population using a model for developing complex interventions in nursing	United States	Case study	Study: a team of HPML^a^ faculty members, a graduate student, a program manager, 2 telehealth experts, and state stakeholders; stakeholders: the TMH^b^ director, BMS^c^ medical director, 2 BMS nurses, the program manager for the state traumatic brain injury waiver, the director of the WV^d^ home- and community-based service program, 1 Medicaid waiver participant advocate, and the chief operations officer.	Multiple chronic conditions	Telehealth
DeHart et al [39], 2022	Benefits and challenges of implementing telehealth in rural settings: a mixed-methods study of behavioral medicine providers	United States	Mixed methods study	17 health care providers	Mental Health	Telehealth
Giroux et al [50], 2022	It’s not one size fits all: a case for how equity-based knowledge translation can support rural and remote communities to optimise virtual health care	Canada	Commentary	General rural population	Equitable access to health care services	VH^e^: hospital in the home
Haque et al [40], 2021	Factors influencing telehealth implementation and use in frontier critical access hospitals: Qualitative study	United States	Qualitative study	36 stakeholders (8 critical access hospitals)	Frontier critical access care	Telehealth
Head et al [51], 2022	Virtual visits for outpatient burn care during the COVID-19 pandemic	United States	Quantitative study	143 patients	Burn care	VH: hospital in the home
Hirko et al [41], 2020	Telehealth in response to the COVID-19 pandemic: Implications for rural health disparities	United States	Commentary	General rural population	Ambulatory practices and triage before hospitalization	Telehealth
Howland et al [42], 2021	Psychiatrist and psychologist experiences with telehealth and remote collaborative care in primary care: a qualitative study	United States	Qualitative study	10 telepsychiatrists and 4 telepsychologists	Mental Health	Telehealth
Jong et al [44], 2019	Enhancing access to care in northern rural communities via telehealth	Canada	Commentary	General rural population	General health care	Telehealth
LeBlanc et al [58], 2020	Patient and provider perspectives on eHealth interventions in Canada and Australia: A scoping review	Australia and Canada	Scoping review	General rural population	General health care	Remote patient monitoring
Thomas et al [48], 2023	Health workforce perceptions on telehealth augmentation opportunities	Australia	Qualitative study	53 health care professionals	Primary health care	Telehealth
Johnston et al [43], 2020	“From the technology came the idea”: safe implementation and operation of a high quality teleradiology model increasing access to timely breast cancer assessment services for women in rural Australia	Australia	Qualitative study	55 service providers	Breast cancer screening	Telehealth
Correale et al [49], 2022	A model to implement standardised virtual care for low back pain amongst a large network of providers in urban and rural settings	Canada	Case study	12 focus groups	Management of lower back pain	VH: hospital in the home
Kocanda et al [45], 2021	Informing telehealth service delivery for cardiovascular disease management: exploring the perceptions of rural health professionals	Australia	Qualitative study	10 health care professionals	Cardiovascular disease	Telehealth
McPherson and Nahon [46], 2021	Telehealth and the provision of pelvic health physiotherapy in regional, rural and remote Australia	Australia	Qualitative study	54 participants	Pelvic health	Telehealth
Allan et al [59], 2021	“This is streets ahead of what we used to do”: staff perceptions of virtual clinical pharmacy services in rural and remote Australian hospitals	Australia	Case study followed by qualitative analysis	15 focus groups (42 nurses, 8 physicians, 12 managers, 4 allied health staff, and 3 pharmacists)	Use of medications in rural and remote NSW^f^ public hospitals	Virtual pharmacy
Nataliansyah et al [47], 2022	Managing innovation: a qualitative study on the implementation of telehealth services in rural emergency departments	United States	Qualitative study	18 participants	Emergency department	Telehealth
Kuperman et al [52], 2018	The virtual hospitalist: a single site implementation bringing hospitalist coverage to critical access hospitals	United States	Case study	General rural population	Emergency and inpatient care at critical access hospitals	VH: hospital in the home
Sitammagari et al [53], 2021	Insights from rapid deployment of a virtual hospital as standard care during the COVID-19 pandemic	United States	Case study	1477 patients	COVID-19	VH: hospital in the home
Vindrola-Padros et al [54], 2021	Remote home monitoring (virtual wards) for confirmed or suspected COVID-19 patients: a rapid systematic review	United Kingdom, United States, Canada, China, the Netherlands, Ireland, Brazil, and Australia	Systematic review	General rural population	COVID-19 positive cases	VH: hospital in the home
Haleem et al [56], 2021	Telemedicine for health care: capabilities, features, barriers, and applications	India	Commentary	General rural population	General health care	Telemedicine
Gray et al [57], 2022	The rapid development of virtual care tools in response to COVID-19: case studies in three Australian health services	Australia	Qualitative study	13 participants	COVID-19	Remote patient monitoring
Bradford et al [37], 2016	Telehealth services in rural and remote Australia: a systematic review of models of care and factors influencing success and sustainability	Australia	Systematic review	General rural population	General health care	Telehealth

^a^HPML: health policy, management, and leadership.

^b^TMH: Take Me Home.

^c^BMS: Bureau for Medical Services.

^d^WV: West Virginia

^e^VH: virtual hospital.

^f^NSW: New South Wales.

**Table 3:** Included gray literature sources.

Authors or sources	Title	Country	Literature type	Health care conditions addressed	Intervention
Shaw and Wilson [71], 2022	RPA virtual hospital proof of concept trial evaluation report 2020–2021	Australia	Web report	Overall Hospital care	VH^a^
Hardy et al [67]	Improving equity of access in rural and regional health through hybrid and connected care	Australia	Web report	General health	Virtual care from home
Microsoft News Center [70], 2022	Remote Australian community harnesses mixed reality and space technologies to deliver better health care	Australia	Web report	General health	VH
Hospital and Healthcare [69], 2021	The virtual hospital	Australia	Web report	General health	VH
Agency for Clinical Innovation [60], 2021	Virtual care in practice	Australia	Web report	General health	Virtual care from home
Department of Health, Australian government [63], 2022	Future focused primary health care: Australia’s Primary Health Care 10 Year Plan 2022-2032	Australia	National strategy	General health	Virtual care from home
Queensland government [66], 2021	Digital strategy for rural and remote healthcare - 10 year plan	Australia	State strategy	General health	Virtual care from home
Northern Territory government of Australia [65]	NT health virtual care strategy	Australia	State strategy	General health	Virtual care from home
Victoria Department of Health [68], 2022	Virtual care standard and guide	Australia	State strategy	General health	Virtual care from home
Department of Health, Australian government [61], 2016	National strategic framework for rural and remote health	Australia	National strategy	General health	Virtual care from home
Department of Infrastructure, Australian government [62], 2022	Better connectivity plan for regional and rural Australia	Australia	Web report	General health	VH
National Rural Health Alliance [64], 2021	Regional telecommunications review 2021	Australia	Telecommunications survey report	General health	Virtual care from home

^a^VH: virtual hospital.

The included 23 peer-reviewed articles were from 9 different countries, with 10 (43%) studies from the United States [38-42,47,51-53,55], 7 (30%) studies from Australia [37,43,45,46,48,57,59], 3 (13%) from Canada [44,49,50], and 1 (4%) from India [56]. Moreover, 1 (4%) study analyzed data from both Australia and Canada [58], while 1 (4%) was a multinational study where data were combined from the United Kingdom, the United States, Canada, China, the Netherlands, Ireland, Brazil, and Australia [54]. The 12 items of gray literature included in this review were all from Australia [60-71].

The selected academic articles covered a wide range of health care issues. Of the 23 peer-reviewed articles, 5 (22%) concentrated on general and primary health care [37,44,48,56,58], 3 (13%) addressed COVID-19 [53,54,57], 2 (9%) focused on emergency and inpatient care [47,52], and 2 (9%) discussed mental health [39,42]. In addition to this, there were 2 (9%) studies discussing equitable access to health care services [50,55] and 1 (4%) study each addressing ambulatory practices and triage [41], breast cancer assessment [43], burn care [51], cardiovascular disease [45], lower back pain [49], multiple chronic conditions [38], pelvic health [46], medication use [59], and frontier critical access care [40]. All 12 (100%) of the 12 items of gray literature addressed general health, focusing mainly on providing equitable health care services virtually [60-71].

In terms of digital technology used for VH implementation, most (20/23, 87%) of the peer-reviewed articles explored the use of videoconferencing [37-39,41-45,47-53,55-59], followed by telephone communication [38,40,41,49,50,53-55,58] and wearable sensors [4,39,41,43,44,54,56,57]. In total, 30% (7/23) of the studies focused on the use of online electronic medical record systems [39,40,49,53,54,57,59], 22% (5/23) of the studies described mobile apps [39,41,48,50,57], while 17% (4/23) of the studies highlighted text messaging for virtual care [41,46,50,57]. Other technologies, such as store and forward (3/23, 13%) [37,43,44], email and chatbot (1/23, 4%) [57], and robotic ultrasonography (1/23, 4%) [44] were also included as supportive VH technologies. Among the 12 gray literature sources, only 2 (17%) reports explicitly addressed virtual care-from-home technologies. Shaw and Wilson [71] discussed the use of videoconferencing, Internet of Medical Things devices, and telephone technology for remote monitoring and directed self-care, while Microsoft News Center [70] concentrated on the use of mixed reality and space technology to support VH implementation. The remaining 10 (83%) gray literature sources holistically discussed the implementation and use of digital care technologies.

### Study Design

Among the 23 peer-reviewed studies, 6 (26%) were case studies [38,49,52,53,55,59] and 1 (4%) was a mixed methods study [39]. In addition, there were 8 (35%) qualitative studies [40,42,43,45-48,57], 1 (4%) quantitative study [51], 1 (4%) scoping review [58], 2 (9%) systematic reviews [37,54], and 4 (17%) low-quality commentaries [41,44,50,56]. Among all included articles, only 2 (9%) reported selection bias [51,53]. Among the 12 gray literature sources, there were 2 (17%) national strategies [61,63], 3 (25%) state-level strategy documents [65,66,68], 6 (50%) web reports [60,62,67,69-71], and 1 (8%) telecommunication survey report [64].

### Clinical Outcomes

Only 7 (30%) academic studies of the 23 peer-reviewed articles and 2 (17%) of the 12 gray literature sources assessed the clinical outcomes of the VHs or virtual care-from-home models.

Findings from existing literature demonstrated that VHs have contributed toward positive clinical outcomes, including specialist medication advice, better medication knowledge, reduced medication errors [59], reduced disease transmission [53,56], minimized disease exposure to staff [53], improved patient safety [57,59], reduced admission and readmission [52,71], lower mortality, shorter LOS, and maximized adherence to clinical best practices [52,54,69].

According to Allan et al [59], VHs facilitated enhanced medication management by enabling specialists to provide tailored advice based on individual patient needs. This personalized approach improved patient understanding and medication adherence. Furthermore, by facilitating direct communication between health care providers and specialists, VHs could help reduce medication errors because specialists could review prescriptions remotely, identify potential errors, and provide immediate feedback or corrections.

VHs also enabled remote consultations, diagnosis, and monitoring, reducing the need for physical interaction between patients and health care staff [53,56]. This reduced the risk of disease transmission, particularly in cases of infectious diseases. Moreover, by minimizing in-person visits, health care staff were exposed to fewer patients, reducing their risk of contracting contagious diseases.

Gray et al [57] and Allan et al [59] emphasized the role of continuous monitoring and timely interventions in enhancing patient safety. Remote monitoring devices and telemedicine consultations enabled early detection of complications, facilitating prompt medical interventions and reducing the risk of adverse events. Through remote monitoring and timely interventions, VHs could also manage chronic conditions more effectively, thereby reducing the frequency of hospital admissions and readmissions [52,71]. In addition, by providing patients with better access to health care resources and support, VHs could prevent exacerbations of chronic conditions, further reducing hospitalizations.

VHs have demonstrated potential in improving patient outcomes and reducing health care costs. By enabling continuous monitoring and timely interventions, VHs contributed to reduced mortality rates, shorter hospital stays, and lower readmission rates. Vindrola-Padros et al [54] reported that virtual wards for patients with COVID-19 had lower mortality rates, with only 29% admissions to the emergency department and <36% readmission rate. Likewise, Kuperman et al [52] reported that the pilot VH recorded a 6% decrease in patient transfers, a 17% decrease in admissions, and a 3.7% decrease in LOS. Furthermore, Head et al [51] discussed that virtual care-from-home service for patients with burn injury had similar clinical outcomes when compared to in-person care. None (0/23, 0%) of the studies reported negative clinical outcomes for VHs.

Despite diverse health care conditions, studies revealed similar clinical outcomes. For instance, Head et al [51] concentrated on burn care, Kuperman et al [52] discussed emergency and inpatient care, and Shaw and Wilson [71] addressed overall hospital care; however, all reported that VHs reduced admission, readmission, and transfer in their respective studies. Likewise, Gray et al [57], addressing virtual pharmacy, and Allan et al [59], discussing COVID-19, discovered that virtual care-from-home solutions improved patient safety.

### Health System Outcomes

A total of 12 (52%) academic studies of the 23 peer-reviewed articles and 3 (25%) of the 12 gray literature sources discussed 9 positive health system outcomes.

The reported outcomes included improved access to care and efficient use of resources [39,52,54,71], reduced patient and health care costs [37,39,48,51,53,56,58], enhanced patient experience and safety [56-58,69], enhanced health care delivery and provider support through trusted workflow and virtual relocation [53,54,58,59], improved process outcomes and enhanced compliance [37,52,54,58,71], enhanced geographic coverage, and better integration of health care services [67,71].

VHs used telemedicine technologies to provide remote health care services, enabling patients to access health care professionals without physical travel and enhancing access to care in underserved areas [39,52,54,71]. Similarly, VHs enhanced health care efficiency by streamlining delivery processes, reallocating resources toward virtual care platforms, reducing physical infrastructure requirements, and resulting in cost savings and improved service delivery.

VHs also lowered patient and health care costs by minimizing the need for in-person visits, hospitalizations, and associated travel expenses. Telemedicine consultations and remote monitoring enabled timely interventions, preventing costly complications and reducing the overall health care expenditure for patients and health care providers. According to Head et al [51], virtual visits resulted in significant savings, including an average reduction of 130 miles in travel distance, 164 minutes of travel time, US $104 in travel costs, and US $81 in forgone wages due to the time saved from not having to travel. None (0/23, 0%) of the articles highlighted negative health care system outcomes.

Furthermore, VHs could offer convenient and personalized health care experiences tailored to patients’ needs because remote consultations and digital health tools empowered patients to actively participate in their care, leading to greater satisfaction and engagement [56-58,69]. In addition, virtual care models prioritized patient safety by minimizing exposure to health care–associated infections and promoting adherence to evidence-based practices. Similarly, by leveraging technology to optimize health care delivery processes and support health care providers in delivering high-quality care, VH facilitators had managed to enable seamless coordination among multidisciplinary teams, improving communication, collaboration, and patient outcomes [53,54,58,59]. The virtual support systems also enhanced provider efficiency and job satisfaction, ultimately enhancing the overall quality of care delivery.

Findings from the literature also revealed that VHs improved health care by standardizing workflows, promoting clinical adherence, and facilitating efficient data management [37,52,54,58,71]. The use of digital health solutions helped improve care coordination and patient outcomes while ensuring regulatory compliance and quality standards in health care delivery.

The findings were consistent across different health care conditions. While Head et al [51], DeHart et al [39], Kuperman et al [52], Shaw and Wilson [71], and Vindrola-Padros et al [54] focused on burn care, mental health, emergency and inpatient, general hospital care, and COVID-19, respectively, their studies collectively highlighted that health care virtualization significantly improved access to care. Furthermore, the Australian-based Royal Prince Alfred VH received positive feedback from patients for the convenience and stress-reducing benefits of receiving care from home [71].

In addition, Hardy et al [67] and Shaw and Wilson [71] reported that VHs offered health care services beyond traditional boundaries, improving accessibility and enabling broader geographic coverage. Telemedicine and remote monitoring enabled patients to have specialized care, reducing disparities in access. This care model also facilitated better integration of health care services by fostering collaboration among providers, improving care transitions, and promoting continuity of care across different settings.

### Patient Perspectives

A total of 8 (35%) of the 23 peer-reviewed articles and 1 (8%) web report of the 12 gray literature sources examined patient perspectives regarding the implementation of VHs and virtual care-from-home solutions.

Allan et al [59] and Correale et al [49] highlighted high patient acceptance and satisfaction with virtual care-from-home services. Similarly, DeHart et al [39] noted increased satisfaction due to time, cost, and travel savings from telehealth services for rural patients. Likewise, Giroux et al [50] disclosed that quality virtual care-from-home enhanced empowerment, improved self-management, and expanded access to culturally appropriate care locally.

Patient involvement in remote care is associated with improved outcomes and satisfaction, as suggested by Vindrola-Padros et al [54]. VHs provided patients with the convenience of receiving treatment locally rather than having to relocate to a secondary care hospital, thus leading to an increased level of satisfaction, as highlighted by Gray et al [57] and Kuperman et al [52]. While none (0/23, 0%) of the studies discussed the negative patient perspectives, LeBlanc et al [58] stated that patient satisfaction was highly dependent on technological proficiency, health care provider readiness, and the quality of care provided virtually.

### Health Care Provider Perspectives

A total of 8 (35%) of the 23 peer-reviewed articles and 1 (8%) of the 12 gray literature sources discussed the provider perspectives on the implementation of VHs and virtual care-from-home solutions.

Positive health care provider perspectives included increased provider satisfaction [43,59], enhanced service delivery [43,59], better collaboration and teamwork [39,49,50,60], and increased efficiency and accessibility [52,56,58,60]. Providers reported that VHs and hospital-in-the-home solutions offered them more flexibility in delivering care and managing their workload. They could engage in telemedicine consultations, remote monitoring, and virtual care delivery, which led to reduced burnout and increased job satisfaction. The convenience and efficiency of virtual care platforms contributed to a more satisfying work environment for health care professionals. Both peer-reviewed articles and gray literature also highlighted that providers reported that VHs improved health care delivery by enabling telemedicine consultations and remote monitoring, thus allowing them to reach a wider patient population, deliver timely interventions, and offer continuous support. Furthermore, the providers stated that the integration of digital health tools allowed for enhanced service quality, accessibility, and patient outcomes.

In addition, health care providers expressed that virtual care platforms allowed them to facilitate collaboration and communication among health care team members, regardless of their physical location [52,56,58,60]. Rural providers could easily consult with specialists, share patient information, and coordinate care plans in real time, promoting interdisciplinary collaboration and teamwork. This seamless exchange of information enhanced care coordination and improved patient outcomes.

Studies by Allan et al [59] and Johnston et al [43] indicated that enhanced service timeliness, greater accessibility, and reduced provider travel time increased provider satisfaction. They also stated that improved workflows and clinic processes and better medication management further contributed to enhanced service delivery.

No patients or health care providers reported negative views of the results.

### Barriers to VH Implementation

Barriers to VH implementation were reported in 21 (91%) of the 23 peer-reviewed articles and 6 (50%) web reports of the 12 gray literature sources.

Poor digital literacy and language barriers among rural patients emerged as significant obstacles, hindering successful VH adoption. Language barriers and a lack of digital skills presented challenges in connecting remote patients with virtual care solutions, contributing to a digital gap [48,50,53,58,71]. The lack of digital skills also presented a substantial obstacle in connecting remote patients with virtual care-from-home solutions [39,57,71]. Poor digital literacy and language barriers among patients often deterred them from engaging in virtual care services [57] and led to a lack of patient commitment to continue using virtual care-from-home services [39]. Furthermore, health care providers’ low adoption and lack of provider knowledge or skills were reported, resulting in resistance to change and workforce constraints, indicating a need for targeted strategies to address these issues [37-40,44,48,53,68,71].

Technological barriers, such as poor technical infrastructure and connectivity [41,45,48-51,53,56,58,64,67,68], limited technical support [39,46,50,57,58,60,68,71], restricted access to digital equipment [38,43,46,49,53,57,60], and legacy and outdated technologies [47,68,71], were also identified as significant barriers to VH implementation for regional and remote communities.

In remote and rural areas, inadequate technical infrastructure and connectivity hindered the effective deployment of VH services [41,45,48-51,53,56,58,64,67,68]. Without reliable internet access and robust communication networks, health care providers and patients faced challenges in accessing telemedicine consultations, remote monitoring, and digital health platforms.

Similarly, the absence of adequate technical support exacerbated challenges associated with VH implementation [39,46,50,57,58,60,68,71]. Health care providers and patients encountered difficulties in troubleshooting technical issues, configuring digital devices, or navigating telemedicine platforms.

Another major technological barrier faced by individuals in remote and underserved communities was the lack of access to digital devices, such as tablets, webcams, or computers, which were essential for participating in virtual care consultations [38,43,46,49,53,57,60]. The digital divide exacerbated disparities in health care access and highlighted the need for initiatives aimed at providing digital equipment to marginalized populations.

Moreover, the presence of legacy and outdated technologies impeded the adoption and effectiveness of VH solutions [47,68,71]. Incompatible systems, outdated software, and obsolete hardware hindered interoperability, data exchange, and user experience.

Several studies reported barriers related to processes of coordination and communication among stakeholders [38,58], cultural neglect for rural communities [66,68], absence of robust governance and effective leadership [42,43,59,68,71], data privacy and security concerns [38,48,49,56,58,69], organizational challenges in relation to resource planning [54,59], operational processes [38,42,60], and limited policies supporting rural community inclusion [45,48,54,57].

According to Davis et al [38] and LeBlanc et al [58], effective coordination and communication among stakeholders were essential for the successful implementation of VHs. Barriers within these processes, such as fragmented communication channels or a lack of standardized protocols, impeded the seamless delivery of care and collaboration among health care providers, patients, and support staff.

In addition, the cultural neglect of rural communities highlighted disparities in health care access and delivery. Policies and initiatives that failed to consider the unique cultural, social, and geographic factors of rural populations exacerbated health care inequities [66,68]. Recognizing and addressing cultural diversity and rural-specific health care needs were essential for ensuring inclusive and patient-centered VH services.

Furthermore, inadequate governance frameworks and leadership support led to ambiguity, resistance to change, and lack of accountability, hindering progress and sustainability [42,43,59,68,71]. Strong leadership and governance mechanisms were essential for guiding strategic planning, resource allocation, and decision-making processes in VH initiatives, and these were fundamental for driving organizational change and ensuring the successful implementation of VHs.

Another critical consideration for VH implementation was data privacy and security concerns, particularly regarding the storage, transmission, and sharing of sensitive patient information [38,48,49,56,58,69]. Failure to address these concerns could impact patient trust, compromise confidentiality, and expose health care organizations to legal and regulatory risks. Robust data privacy policies, secure information systems, and adherence to industry standards were considered essential for safeguarding patient data and maintaining confidentiality.

Organizational challenges, such as inadequate resource planning and inefficient operational processes, impeded the scalability and sustainability of VHs [54,59]. Limited resources, workforce shortages, and inefficient workflows hindered the delivery of timely and quality care, affecting patient outcomes and satisfaction. Strategic resource allocation, process optimization, and continuous quality improvement were necessary for overcoming organizational barriers and enhancing operational efficiency in VHs.

In addition, limited policies supporting rural community inclusion also affected the implementation of equitable VH services [45,48,54,57]. The absence of supportive policies may have limited funding opportunities, regulatory incentives, and infrastructure development for VHs in rural areas. Advocacy for policies that prioritized rural community inclusion, addressed health care disparities, and incentivized VH adoption were essential for promoting equitable access to health care services.

Furthermore, a few (4/35, 11%) studies identified financial sustainability challenges [47,57,60,69] and the unfair distribution of funds among different health care settings [42,47,57].

### Facilitators of VH Initiatives

Facilitators for the successful implementation of VHs were identified in 19 (83%) of the 23 peer-reviewed articles and 7 (58%) web reports of the 12 gray literature sources.

The literature strongly suggested that key facilitators included clinical leadership and advocacy [37,48,71] together with staff and patient enrichment and training [40,42,43,46,47,52,54,59,71], emphasizing the importance of enhancing patient and provider capabilities for successful VH implementation. The Australian federal and state governments are investing in digital literacy upskilling programs to empower patients to self-manage symptoms and navigate VHs confidently [67,68].

Few other facilitators were recognized as a way to mitigate barriers such as collaboration and partnerships [43,48,57,59,71], effective communication [48,49,52,65], purposeful planning and development [37,60,65,71], a comprehensive governance structure [60,65,71], optimized organizational structure [38,43,49,53], iterative system implementation [59,65,71], and supportive government policies [37,51,67,69].

Collaboration and partnerships among stakeholders, including health care providers, government agencies, technology vendors, and community organizations, were instrumental in overcoming barriers and driving the adoption of VHs [43,48,57,59,71]. By fostering collaboration, organizations could leverage collective expertise, resources, and networks to address challenges; share best practices; and promote innovation in virtual care delivery. Likewise, effective communication strategies were essential for ensuring clear understanding, alignment, and engagement among stakeholders involved in VH implementation. Open and transparent communication channels facilitated knowledge sharing, stakeholder engagement, and problem-solving, enabling organizations to address concerns, manage expectations, and drive consensus toward common goals [48,49,52,65].

Purposeful planning and development also incorporated strategic initiatives aimed at designing, implementing, and optimizing VHs [37,60,65,71]. By adopting a systematic approach to planning, organizations could anticipate challenges, identify opportunities, and develop robust strategies for addressing barriers and achieving desired outcomes in virtual care delivery. Similarly, a comprehensive governance structure provided the framework for guiding decision-making, accountability, and oversight in VH initiatives [60,65,71]. Clear governance mechanisms, policies, and procedures ensured alignment with organizational goals, regulatory requirements, and industry standards while also promoting transparency, efficiency, and stakeholder engagement.

In addition, an optimized organizational structure will align roles, responsibilities, and workflows to support the effective delivery of VH services [38,43,49,53]. By streamlining processes, clarifying responsibilities, and fostering collaboration, organizations can enhance efficiency, agility, and responsiveness in virtual care delivery, ultimately improving patient outcomes and satisfaction. Likewise, iterative system implementation will involve continuous assessment, refinement, and optimization of VH services and processes. By adopting an iterative approach, organizations can adapt to evolving needs, address emerging challenges, and incorporate feedback from stakeholders, thereby enhancing the effectiveness, usability, and sustainability of virtual care solutions [59,65,71].

Moreover, supportive government policies created an enabling environment for VH implementation by providing regulatory intelligibility, funding support, and incentives for innovation. Policies that promoted telemedicine reimbursement, infrastructure development, workforce training, and data privacy enhanced the viability and scalability of virtual care initiatives while also fostering collaboration and alignment among stakeholders across the health care ecosystem [37,51,67,69].

These facilitators highlighted the importance of strategic planning, clear communication, and collaborative efforts involving various stakeholders to overcome process-related challenges and ensure successful VH deployment.

Investment in *technical infrastructure* [59,60,65,67,68,70], quality, user-friendly and affordable technology [53,59,60,67,68], and technical outreach and support services [43,58,68] were noted as enablers for VH initiatives.

Robust technical infrastructure formed the foundation for VHs, facilitating seamless communication, data exchange, and service delivery [59,60,65,67,68,70]. Hence, investment in infrastructure, such as high-speed internet connectivity, secure data storage, and interoperable systems, was essential for ensuring reliable and efficient virtual care delivery. Without adequate infrastructure, VHs would face connectivity issues, data security risks, and operational inefficiencies, hindering their effectiveness in reaching and serving rural patients.

Furthermore, access to quality, user-friendly, and affordable technology was critical for enhancing the accessibility and usability of VHs [53,59,60,67,68]. User-friendly interfaces, intuitive design, and affordability ensured that health care providers and patients could easily navigate and use virtual care platforms. Quality technology solutions also contributed to positive user experiences, engagement, and satisfaction, ultimately improving the effectiveness and adoption of VHs.

Similarly, technical outreach and support services played a vital role in assisting health care providers and patients in navigating and troubleshooting virtual care technology services [43,58,68]. These services might include training programs, help desks, and technical assistance teams that provide guidance, education, and troubleshooting support. By offering ongoing technical support, organizations could empower users to effectively use VH platforms, address technical issues, and maximize the benefits of remote care delivery.

These facilitators emphasized the importance of a robust technical foundation, user-friendly interfaces, and support services to overcome technological challenges and enhance the acceptance of VHs within regional and remote communities.

*Sustainable financing* also emerged as a crucial facilitator, emphasizing the importance of permanent insurance reimbursement solutions and grant funding to support and expand the reach of VH services [38,47,53].

## Discussion

### Principal Findings

This review demonstrated clinical and health system outcomes of VHs in remote and rural health settings. It addresses the current evidence gap regarding the impact of VHs for regional and rural populations on clinical, health system, patient, and provider outcomes, indicating that VHs significantly enhance clinical effectiveness, regardless of population demographics.

In terms of clinical effectiveness, this review reported positive outcomes against key indicators commonly used to evaluate health care interventions, such as mortality rates, readmission rates, LOS, and improvement in clinical indicators [71]. These findings are consistent with those of Norman et al [17], who reported similar benefits from hospital-at-home care for older individuals, such as shorter LOS and reduced readmission rates. However, it should be noted that this review does not examine the impact of VHs on chronic condition management, a critical area for evaluating the overall effectiveness of VHs. Further research is needed to explore how VHs can better support chronic disease management and improve long-term health outcomes.

Furthermore, this study highlights the health system benefits of VHs, noting their potential to reduce patient and health care expenditures for various conditions, including general health, mental health, burn care, and infectious diseases such as COVID-19. The analysis correlates with key metrics such as quality care, equitable access, cost management, and population health improvement, affirming that VHs significantly enhance health system outcomes in regional and remote communities.

Moreover, the health system outcomes identified in this review align with those observed in urban populations, as reported by Bidoli et al [13] and Snoswell et al [72]. Evidence indicates that VHs positively impact health system outcomes by improving efficiency, accessibility, and resource management, thus proving to be a valuable addition to contemporary health care delivery.

The findings also reveal that positive patient and provider perspectives are contingent on adequate internet connectivity, user-friendly technology, sufficient training, and enhanced collaboration between urban and rural health care providers. A comparison with the studies by Denny and Hill [73], who evaluated patient and health care worker perspectives of virtual care delivery for patients with cystic fibrosis in a suburban setting, and Babaei et al [18], who assessed barriers to and facilitators of virtual care, revealed similar findings. Both studies support the argument that high patient and provider satisfaction heavily depends on adequate internet connectivity, effective technology, and robust technical support systems.

Beyond the findings of this study, it is worth noting that VHs also have the transformative potential in mitigating climate change while maintaining effective patient care. King et al [74] revealed a 99.37% reduction in carbon emissions per appointment compared to in-person visits. Likewise, Thiel et al [75] demonstrated that telemedicine reduced greenhouse gas emissions by nearly 17,000 metric tons in 2021, equivalent to the annual energy use of 2100 homes, by replacing in-person visits with virtual appointments.

**Figure 3:**
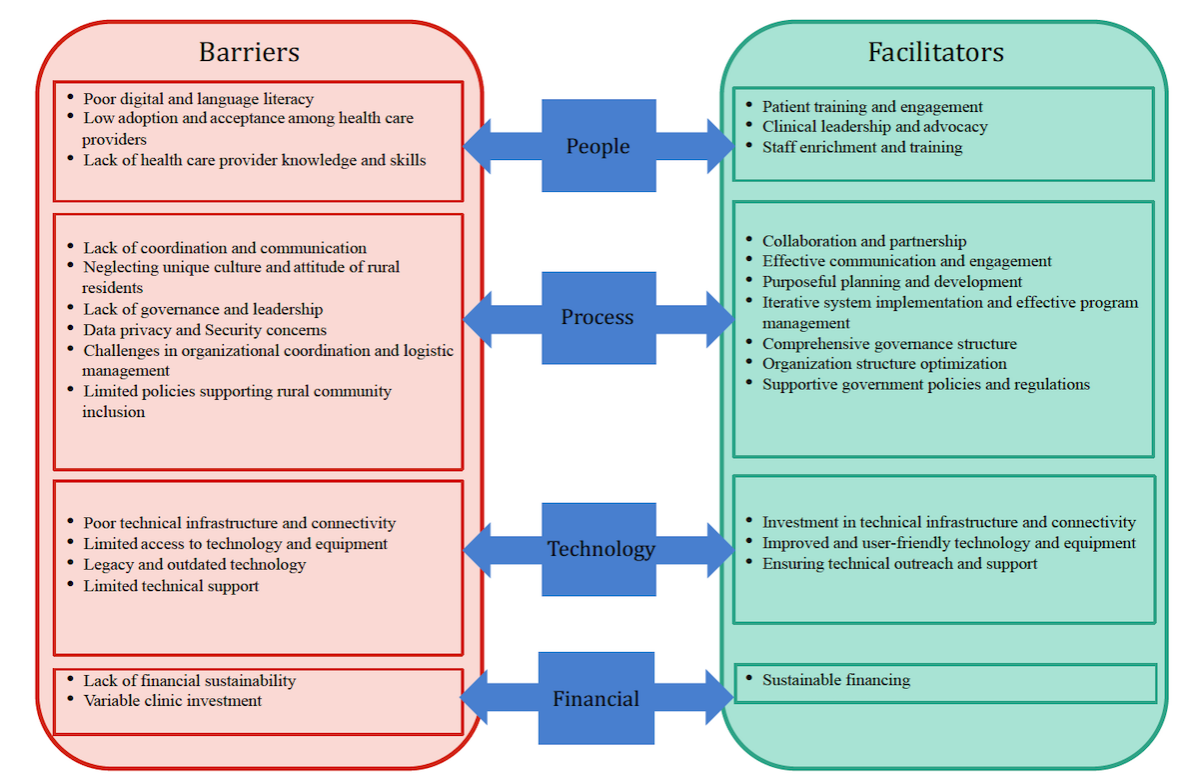
Barriers to and facilitators of virtual hospital initiatives.

Despite the numerous benefits of VHs for rural and remote health care, their implementation often faces significant challenges. These challenges can be categorized into 4 main themes: people, process, technology, and finance (Figure 3). Effectively addressing barriers in these areas requires a multifaceted approach, including digital literacy and enrichment, stakeholder collaboration, a patient-centered approach, sustainable funding structures, and the application of smart technologies. By implementing these strategies, regional and remote communities can gain better access to quality health care, potentially improving clinical and health system outcomes and enhancing satisfaction among rural patients and providers.

Addressing these multifaceted challenges is crucial for the successful implementation of VHs in regional and remote health care settings. The identified facilitators provide a road map for strategic interventions and investments to overcome barriers, thereby enhancing the effectiveness of VHs in improving health care accessibility and delivery in underserved areas.

### Recommendations for Developing VHs

The synthesis of studies on the development of VHs in regional and remote areas has led to 4 key recommendations. First, successful VH implementation relies heavily on stakeholder collaboration and patient-centered approaches. Engaging a diverse range of stakeholders, including community members and health care providers, ensures that VHs align with the needs and preferences of all involved. Key elements include collaborative engagement, health care professional advocacy, seamless communication policies, patient-centered design, and culturally responsive models. These strategies foster trust, improve coordination, and ensure inclusivity among and respect for diverse populations.

Second, achieving digital equity and securing sustainable funding are critical for the success of VHs. Implementing robust governance structures and advisory committees to oversee VH operations ensures coordination, safety, and quality standards. Policy-driven organizational transformation enables VHs to adapt to evolving health care needs while leveraging government grants to provide essential financial support, addressing infrastructure needs and operational deficits.

Third, investing in sustainable infrastructure and advancing health care technology are vital for enhancing VH services. Recommendations include developing resilient infrastructure to support VH technology, advancing integrated health care systems, empowering remote care staff with the necessary tools and training, building robust digital foundations through iterative development, and implementing stringent privacy and security measures to protect patient data.

Finally, enhancing digital literacy among health care providers and patients is crucial for the seamless implementation of VHs. Continuous training and involvement of health care providers build trust and competence in using new technologies, while educating patients about virtual care technologies improves acceptance and engagement.

Overall, successful VH implementation in regional and remote regions requires a holistic approach that considers stakeholder collaboration, a patient-centered approach, digital equity, smart technology application, and digital literacy enhancement. These recommendations provide a comprehensive framework for developing and sustaining VHs in regional and remote health care settings.

### Limitations and Future Research Direction

This review encountered several limitations. First, the restricted search period, which concluded in March 2023, limited the capture of more recent developments and emerging insights. However, the unique focus on the remote and rural context offers timeless relevance to the baseline understanding of regional and remote VH implementation globally during the COVID-19 pandemic. This study provides a valuable foundation for future comparative research on VH implementation across rural and urban settings. Second, the absence of studies reporting negative outcomes restricts our understanding of the potential risks and drawbacks associated with the development of VHs for remote and regional communities. This lack of balanced reporting may lead to an overly optimistic view of VH implementation. In addition, as a rapid review, this study faced typical limitations, such as a constrained time frame and reliance on English language sources, potentially excluding relevant studies and resulting in a less comprehensive literature search. While efficient, the rapid review methodology offers less depth compared to systematic reviews, potentially limiting the robustness of conclusions. Finally, this review insufficiently emphasized the broader environmental implications of VHs, particularly their demonstrated potential to reduce carbon emissions through decreased patient travel and resource consumption. This oversight limits the discussion of their potential as a tool for environmental sustainability. Finally, despite the authors’ efforts to avoid using region-specific terms in the gray literature search, it is important to note that the use of the Google search engine in this study may have contributed to geographic bias, leading to an emphasis on Australian-focused gray literature sources.

Future research should focus on evaluating both positive and negative outcomes of VHs compared to inpatient care, using robust methodologies, such as randomized controlled trials or matched cohort studies. Such research would provide a comprehensive evaluation of the clinical and health system outcomes of VHs, facilitating direct comparisons to traditional inpatient care. Moreover, there is a pressing need for qualitative research involving interviews with key stakeholders engaged in successful VH projects. Analyzing these interviews would yield valuable insights into the challenges, successes, and lessons learned from specific projects, enabling the development of well-informed recommendations for future VH initiatives. In addition, research should investigate how implementing VH services in rural and remote areas could contribute to climate change mitigation through reduced health care–related travel and resource consumption.

### Conclusions

Research into VHs is continuously evolving due to developments in regulations, digital advancements, communication tools, and remote monitoring capabilities. By bringing together information across different disciplines, including health care, technology, and policies, this evidence-based rapid review aids in effectively strategizing VH deployment so that the benefits can be realized by regional and remote communities.

This review suggests that VHs provide a promising solution to bridge health care gaps in regional and remote communities by enhancing clinical effectiveness, improving health system outcomes, and increasing patient and provider satisfaction. This review also supports the positive contribution of VHs on equitable health care access and addresses the existing evidence gap within the context of regional and rural health care.

To sum up, this rapid review highlights the barriers, mitigating facilitators, and recommendations that can serve as key strategic pillars to developing VHs so that clinical outcomes, health system performance, and satisfaction among patients and health care providers are enhanced within regional and remote communities.

## Appendix

Multimedia Appendix 1 PRISMA checklist.

Multimedia Appendix 2 Search strategy.

Multimedia Appendix 3 Quality criteria checklist.

Multimedia Appendix 4 Data extraction table.

Multimedia Appendix 5 Overall concept matrix.

## Abbreviations

LOS: length of stay
PICO: population, intervention, comparison, and outcome
PRISMA: Preferred Reporting Items for Systematic Reviews and Meta-Analyses
RPM: Remote Patient Monitoring
VH: virtual hospital

## References

[1] Dixit SK, Sambasivan M (2018). A review of the Australian healthcare system: a policy perspective. SAGE Open Med.

[2] Rural and remote health. Australian Institute of Health and Welfare, Australian Government.

[3] Rechel B, Džakula A, Duran A, Fattore G, Edwards N, Grignon M, Haas M, Habicht T, Marchildon GP, Moreno A, Ricciardi W, Vaughan L, Smith TA (2016). Hospitals in rural or remote areas: an exploratory review of policies in 8 high-income countries. Health Policy.

[4] Thomas SL, Wakerman J, Humphreys JS (2015). Ensuring equity of access to primary health care in rural and remote Australia - what core services should be locally available?. Int J Equity Health.

[5] About rural health. Centers for Disease Control and Prevention (CDC).

[6] Ghafouri Fard M, Hasankhani HH (2015). Virtual hospital: a new approach in education and treatment. J Med Edu Dev.

[7] Hesse BW, Ahern D, Ellison M, Aronoff-Spencer E, Vanderpool RC, Onyeije K, Gibbons MC, Mullett TW, Chih MY, Attencio V, Patterson G, Boten J, Hartshorn C, Bartolome B, Gorscak K, McComsey M, Hubenko A, Huang B, Baker C, Norman D (2020). Barn-raising on the digital frontier: the L.A.U.N.C.H. collaborative. J Appalach Health.

[8] Talevski J, Semciw AI, Boyd JH, Jessup RL, Miller SM, Hutton J, Lawrence J, Sher L (2024). From concept to reality: a comprehensive exploration into the development and evolution of a virtual emergency department. J Am Coll Emerg Physicians Open.

[9] Katie A How Ochsner Health’s RPM pilot improved outcomes for patients with diabetes, hypertension. MedCity News.

[10] Martinez KA, Deshpande A, Stanley E, Rothberg MB (2025). Antibiotic prescribing for respiratory tract infections in urgent care: a comparison of in-person and virtual settings. Clin Infect Dis.

[11] WHO-ITU global standard for accessibility of telehealth services. World Health Organization.

[12] Digital health: a framework for healthcare transformation. Healthcare Information and Management Systems Society (HIMSS).

[13] Bidoli C, Pegoraro V, Dal Mas F, Bagnoli C, Bert F, Bonin M, Butturini G, Cobianchi L, Cordiano C, Minto G, Pilerci C, Stocco P, Zantedeschi M, Campostrini S (2025). Virtual hospitals: the future of the healthcare system? An expert consensus. J Telemed Telecare.

[14] Gunsilius CZ, Heffner J, Bruinsma S, Corinha M, Cortinez M, Dalton H, Duong E, Lu J, Omar A, Owen LL, Roarr BN, Tang K, Petzschner FH (2024). SOMAScience: a novel platform for multidimensional, longitudinal pain assessment. JMIR Mhealth Uhealth.

[15] Digital health. World Health Organization.

[16] Catallo C, Chung-Lee L (2022). How has COVID-19 changed the way we do virtual care? A scoping review protocol. Healthcare (Basel).

[17] Norman G, Bennett P, Vardy ER (2023). Virtual wards: a rapid evidence synthesis and implications for the care of older people. Age Ageing.

[18] Babaei N, Zamanzadeh V, Valizadeh L, Lotfi M, Samad-Soltani T, Kousha A, Avazeh M (2023). A scoping review of virtual care in the health system: infrastructures, barriers, and facilitators. Home Health Care Serv Q.

[19] rpavirtual - a new way of caring. Sydney Local Health District.

[20] Duska A Mayo Clinic launches advanced care at home model of care. Mayo Clinic.

[21] Virtual service expanding to relieve hospital pressures. Premier of Victoria.

[22] O'Kane G (2020). Telehealth-Improving access for rural, regional and remote communities. Aust J Rural Health.

[23] (2018). Australia’s national digital health strategy. Australian Digital Health Agency.

[24] Dhala A, Sasangohar F, Kash B, Ahmadi N, Masud F (2020). Rapid implementation and innovative applications of a virtual intensive care unit during the COVID-19 pandemic: case study. J Med Internet Res.

[25] Gajarawala SN, Pelkowski JN (2021). Telehealth benefits and barriers. J Nurse Pract.

[26] Alison B (2021). Rapid review of virtual care. Consumers Health Forum of Australia.

[27] Armstrong CM, Wilck NR, Murphy J, Herout J, Cone WJ, Johnson AK, Zipper K, Britz B, Betancourt-Flores G, LaFleur M, Vetter B, Dameron B, Frizzell N (2022). Results and lessons learned when implementing virtual health resource centers to increase virtual care adoption during the COVID-19 pandemic. J Technol Behav Sci.

[28] Richards D, Caldwell P (2018). Improving health outcomes sooner rather than later via an interactive website and virtual specialist. IEEE J Biomed Health Inform.

[29] Summerfelt WT, Sulo S, Robinson A, Chess D, Catanzano K (2015). Scalable hospital at home with virtual physician visits: pilot study. Am J Manag Care.

[30] Tricco AC, Langlois EV, Straus SE (2017). Rapid reviews to strengthen health policy and systems: a practical guide. World Health Organization.

[31] Page MJ, McKenzie JE, Bossuyt PM, Boutron I, Hoffmann TC, Mulrow CD, Shamseer L, Tetzlaff JM, Akl EA, Brennan SE, Chou R, Glanville J, Grimshaw JM, Hróbjartsson A, Lalu MM, Li T, Loder EW, Mayo-Wilson E, McDonald S, McGuinness LA, Stewart LA, Thomas J, Tricco AC, Welch VA, Whiting P, Moher D (2021). The PRISMA 2020 statement: an updated guideline for reporting systematic reviews. BMJ.

[32] Huang X, Lin J, Demner-Fushman D (2006). Evaluation of PICO as a knowledge representation for clinical questions. AMIA Annu Symp Proc.

[33] Badampudi D, Wohlin C, Petersen K (2015). Experiences from using snowballing and database searches in systematic literature studies. Proceedings of the 19th International Conference on Evaluation and Assessment in Software Engineering.

[34] Covidence systematic review software. Veritas Health Innovation.

[35] Duval D, Pearce-Smith N, Palmer JC, Sarfo-Annin JK, Rudd P, Clark R (2023). Critical appraisal in rapid systematic reviews of COVID-19 studies: implementation of the Quality Criteria Checklist (QCC). Syst Rev.

[36] Thomas J, Harden A (2008). Methods for the thematic synthesis of qualitative research in systematic reviews. BMC Med Res Methodol.

[37] Bradford NK, Caffery LJ, Smith AC (2016). Telehealth services in rural and remote Australia: a systematic review of models of care and factors influencing success and sustainability. Rural Remote Health.

[38] Davis SM, Jones A, Jaynes ME, Woodrum KN, Canaday M, Allen L, Mallow JA (2020). Designing a multifaceted telehealth intervention for a rural population using a model for developing complex interventions in nursing. BMC Nurs.

[39] DeHart D, King LB, Iachini AL, Browne T, Reitmeier M (2022). Benefits and challenges of implementing telehealth in rural settings: a mixed-methods study of behavioral medicine providers. Health Soc Work.

[40] Haque SN, DeStefano S, Banger A, Rutledge R, Romaire M (2021). Factors influencing telehealth implementation and use in frontier critical access hospitals: qualitative study. JMIR Form Res.

[41] Hirko KA, Kerver JM, Ford S, Szafranski C, Beckett J, Kitchen C, Wendling AL (2020). Telehealth in response to the COVID-19 pandemic: implications for rural health disparities. J Am Med Inform Assoc.

[42] Howland M, Tennant M, Bowen DJ, Bauer AM, Fortney JC, Pyne JM, Shore J, Cerimele JM (2021). Psychiatrist and psychologist experiences with telehealth and remote collaborative care in primary care: a qualitative study. J Rural Health.

[43] Johnston K, Smith D, Preston R, Evans R, Carlisle K, Lengren J, Naess H, Phillips E, Shephard G, Lydiard L, Lattimore D, Larkins S (2020). "From the technology came the idea": safe implementation and operation of a high quality teleradiology model increasing access to timely breast cancer assessment services for women in rural Australia. BMC Health Serv Res.

[44] Jong M, Mendez I, Jong R (2019). Enhancing access to care in northern rural communities via telehealth. Int J Circumpolar Health.

[45] Kocanda L, Fisher K, Brown LJ, May J, Rollo ME, Collins CE, Boyle A, Schumacher TL (2021). Informing telehealth service delivery for cardiovascular disease management: exploring the perceptions of rural health professionals. Aust Health Rev.

[46] McPherson K, Nahon I (2021). Telehealth and the provision of pelvic health physiotherapy in regional, rural and remote Australia. ANZ Cont J.

[47] Nataliansyah MM, Merchant KA, Croker JA, Zhu X, Mohr NM, Marcin JP, Rahmouni H, Ward MM (2022). Managing innovation: a qualitative study on the implementation of telehealth services in rural emergency departments. BMC Health Serv Res.

[48] Thomas LT, Lee CM, McClelland K, Nunis G, Robinson S, Norman R (2023). Health workforce perceptions on telehealth augmentation opportunities. BMC Health Serv Res.

[49] Correale MR, Soever LJ, Rampersaud YR (2022). A model to implement standardized virtual care for low back pain amongst a large network of providers in urban and rural settings. J Prim Care Community Health.

[50] Giroux EE, Hagerty M, Shwed A, Pal N, Huynh N, Andersen T, Banner D (2022). It's not one size fits all: a case for how equity-based knowledge translation can support rural and remote communities to optimize virtual health care. Rural Remote Health.

[51] Head WT, Garcia D, Mukherjee R, Kahn S, Lesher A (2022). Virtual visits for outpatient burn care during the COVID-19 pandemic. J Burn Care Res.

[52] Kuperman EF, Linson EL, Klefstad K, Perry E, Glenn K (2018). The virtual hospitalist: a single-site implementation bringing hospitalist coverage to critical access hospitals. J Hosp Med.

[53] Sitammagari K, Murphy S, Kowalkowski M, Chou S, Sullivan M, Taylor S, Kearns J, Batchelor T, Rivet C, Hole C, Hinson T, McCreary P, Brown R, Dunn T, Neuwirth Z, McWilliams A (2021). Insights from rapid deployment of a "virtual hospital" as standard care during the COVID-19 pandemic. Ann Intern Med.

[54] Vindrola-Padros C, Singh KE, Sidhu MS, Georghiou T, Sherlaw-Johnson C, Tomini SM, Inada-Kim M, Kirkham K, Streetly A, Cohen N, Fulop NJ (2021). Remote home monitoring (virtual wards) for confirmed or suspected COVID-19 patients: a rapid systematic review. EClinicalMedicine.

[55] AlDossary S, Martin-Khan MG, Bradford NK, Armfield NR, Smith AC (2017). The development of a telemedicine planning framework based on needs assessment. J Med Syst.

[56] Haleem A, Javaid M, Singh RP, Suman R (2021). Telemedicine for healthcare: capabilities, features, barriers, and applications. Sens Int.

[57] Gray K, Chapman W, Khan UR, Borda A, Budge M, Dutch M, Hart GK, Gilbert C, Wani TA (2022). The rapid development of virtual care tools in response to COVID-19: case studies in three Australian health services. JMIR Form Res.

[58] LeBlanc M, Petrie S, Paskaran S, Carson D, Peters P (2020). Patient and provider perspectives on eHealth interventions in Canada and Australia: a scoping review. Rural Remote Health.

[59] Allan J, Webster E, Chambers B, Nott S (2021). "This is streets ahead of what we used to do": staff perceptions of virtual clinical pharmacy services in rural and remote Australian hospitals. BMC Health Serv Res.

[60] Virtual care in practice. Agency for Clinical Innovation.

[61] National strategic framework for rural and remote health. Department of Health, Australian Government.

[62] Better connectivity plan for regional and rural Australia. Department of Infrastructure, Regional Development, Communications and the Arts, Australian Government.

[63] Australia’s primary health care 10 year plan 2022–2032. Department of Health, Australian Government.

[64] Regional telecommunications review 2021. National Rural Health Alliance.

[65] NT health virtual care strategy. Northern Territory Government of Australia.

[66] Digital strategy for rural and remote healthcare - 10 year plan. Queensland Government.

[67] Hardy I, Rufus V, Jeyachandran A, Curran J Improving equity of access in rural and regional health through hybrid and connected care. PwC.

[68] Virtual care standard and guide. Victoria Department of Health.

[69] The virtual hospital. Hospital and Healthcare.

[70] Microsoft News Center Remote Australian community harnesses mixed reality and space technologies to deliver better healthcare. Microsoft Australia.

[71] Shaw M, Wilson A (2022). RPA virtual hospital proof of concept trial: evaluation report February 2020 to January 2021. rpavirtual.

[72] Snoswell CL, Taylor ML, Comans TA, Smith AC, Gray LC, Caffery LJ (2020). Determining if telehealth can reduce health system costs: scoping review. J Med Internet Res.

[73] Denny CF, Hill U (2022). P200 A virtual age? Evaluating the patient and healthcare worker perspective on virtual clinic delivery for patients with Cystic Fibrosis (CF) and non CF bronchiectasis (nCFB) at a specialist cardio-thoracic hospital. BMJ J.

[74] King J, Poo SX, El-Sayed A, Kabir M, Hiner G, Olabinan O, Colwill M, Ayubi H, Shakweh E, Kronsten VT, Kader R, Hayee B, GLINT Research Network (2023). Towards NHS Zero: greener gastroenterology and the impact of virtual clinics on carbon emissions and patient outcomes. A multisite, observational, cross-sectional study. Frontline Gastroenterol.

[75] Thiel CL, Mehta N, Sejo CS, Qureshi L, Moyer M, Valentino V, Saleh J (2023). Telemedicine and the environment: life cycle environmental emissions from in-person and virtual clinic visits. NPJ Digit Med.

