# Supplementary material for: Advancing Regional and Remote Health Care With Virtual Hospital Implementation: Rapid Review
Source: JMIR Hum Factors. 2025 Jun 3;12:e64582. doi: 10.2196/64582 (PMC12174879; doi:10.2196/64582)
Supplement: Multimedia Appendix 1 [file humanfactors_v12i1e64582_app1.docx]

| **Section and Topic** | **Item #** | **Checklist item** | **Description/Location** |
| --- | --- | --- | --- |
| **TITLE** | | |  |
| Title | 1 | Identify the report as a systematic review. | The paper is a rapid review (identified in title and description) with certain components of PRISMA checklist expedited |
| **ABSTRACT** | | |  |
| Abstract | 2 | See the PRISMA 2020 for Abstracts checklist. | Structured abstract written as per PRISMA and JMIR guidelines at the start of the paper |
| **INTRODUCTION** | | |  |
| Rationale | 3 | Describe the rationale for the review in the context of existing knowledge. | The background sub-section of the introduction describes the rationale for the review while paragraph 1 of research focus and aim sub-section advocates the need for this review in context of existing knowledge. |
| Objectives | 4 | Provide an explicit statement of the objective(s) or question(s) the review addresses. | Research objectives are discussed in the third paragraph of research focus and aims sub-section of the introduction section. |
| **METHODS** | | |  |
| Eligibility criteria | 5 | Specify the inclusion and exclusion criteria for the review and how studies were grouped for the syntheses. | Specified in the ‘inclusion and exclusion criteria’ sub-section of the methods section. |
| Information sources | 6 | Specify all databases, registers, websites, organisations, reference lists and other sources searched or consulted to identify studies. Specify the date when each source was last searched or consulted. | Specified in ‘Screening and Study Selection’ subsection of the methods section. Databases and search dates are also included. |
| Search strategy | 7 | Present the full search strategies for all databases, registers and websites, including any filters and limits used. | Specified in the ‘Search Strategy’ sub-section of the methods section. |
| Selection process | 8 | Specify the methods used to decide whether a study met the inclusion criteria of the review, including how many reviewers screened each record and each report retrieved, whether they worked independently, and if applicable, details of automation tools used in the process. | This is discussed in ‘Screening and Study Selection’ sub-section of the Methods section . The study selection described, including title/abstract screening and use of Covidence software. |
| Data collection process | 9 | Specify the methods used to collect data from reports, including how many reviewers collected data from each report, whether they worked independently, any processes for obtaining or confirming data from study investigators, and if applicable, details of automation tools used in the process. | Described in and ‘Data Extraction and Knowledge Synthesis’ sub-section of the methods section. |
| Data items | 10a | List and define all outcomes for which data were sought. Specify whether all results that were compatible with each outcome domain in each study were sought (e.g. for all measures, time points, analyses), and if not, the methods used to decide which results to collect. | Study characteristics section from result section, especially table 2 and 3 provides provides list of outcomes e.g. technology intervention and health condition. |
|  | 10b | List and define all other variables for which data were sought (e.g. participant and intervention characteristics, funding sources). Describe any assumptions made about any missing or unclear information. | Study characteristics and study design sub-sections from Results, especially table 2 and 3 provides list of other variables (design, clinical outcomes, country). |
| Study risk of bias assessment | 11 | Specify the methods used to assess risk of bias in the included studies, including details of the tool(s) used, how many reviewers assessed each study and whether they worked independently, and if applicable, details of automation tools used in the process. | Data extraction and knowledge synthesis subsection describes the use of Quality Criteria Checklist (QCC) used for quality assessment (QCC provided in multimedia appendix 2). |
| Effect measures | 12 | Specify for each outcome the effect measure(s) (e.g. risk ratio, mean difference) used in the synthesis or presentation of results. | Not applicable (qualitative synthesis). |
| Synthesis methods | 13a | Describe the processes used to decide which studies were eligible for each synthesis (e.g. tabulating the study intervention characteristics and comparing against the planned groups for each synthesis (item #5)). | The data extraction and knowledge synthesis subsection describe the use of MS Excel based data extraction template, concept matrix and thematic synthesis methods to identify recurring patterns, emerging trends, and insights into common recommendations for developing virtual hospitals and their related barriers and facilitators. Refer Multimedia Appendix 4. |
|  | 13b | Describe any methods required to prepare the data for presentation or synthesis, such as handling of missing summary statistics, or data conversions. | Expedited/Not required especially as the review involves qualitative synthesis. |
|  | 13c | Describe any methods used to tabulate or visually display results of individual studies and syntheses. | Data presented in tables and figures as presented in the results section. |
|  | 13d | Describe any methods used to synthesize results and provide a rationale for the choice(s). If meta-analysis was performed, describe the model(s), method(s) to identify the presence and extent of statistical heterogeneity, and software package(s) used. | This is discussed in ‘Data Extraction and Knowledge Synthesis’ sub-section from Methods Section. |
|  | 13e | Describe any methods used to explore possible causes of heterogeneity among study results (e.g. subgroup analysis, meta-regression). | Not applicable (qualitative synthesis). |
|  | 13f | Describe any sensitivity analyses conducted to assess robustness of the synthesized results. | Not applicable (qualitative synthesis). |
| Reporting bias assessment | 14 | Describe any methods used to assess risk of bias due to missing results in a synthesis (arising from reporting biases). | Expedited (rapid review), independent review of other authors/researchers. |
| Certainty assessment | 15 | Describe any methods used to assess certainty (or confidence) in the body of evidence for an outcome. | Expedited (rapid review), other authors, apart from lead author involved in forming consensus and review qualitative synthesis |
| **RESULTS** | | |  |
| Study selection | 16a | Describe the results of the search and selection process, from the number of records identified in the search to the number of studies included in the review, ideally using a flow diagram. | Results: PRISMA diagram provided in figure 2. |
|  | 16b | Cite studies that might appear to meet the inclusion criteria, but which were excluded, and explain why they were excluded. | Refer to Multimedia Appendix 5. Excluded Studies Reasons. |
| Study characteristics | 17 | Cite each included study and present its characteristics. | Refer to table 2 and 3 of the study characteristics sub-section from Results. |
| Risk of bias in studies | 18 | Present assessments of risk of bias for each included study. | Risk of bias results reported using Quality Criteria Checklist in the multimedia appendix 2. |
| Results of individual studies | 19 | For all outcomes, present, for each study: (a) summary statistics for each group (where appropriate) and (b) an effect estimate and its precision (e.g. confidence/credible interval), ideally using structured tables or plots. | The study characteristics and study design sub-section from result discusses this. Data extraction table also provided as multimedia appendix 6. |
| Results of syntheses | 20a | For each synthesis, briefly summarise the characteristics and risk of bias among contributing studies. | Discussed in Study design sub-section and in the Quality Criteria Checklist (multimedia appendix 2). |
|  | 20b | Present results of all statistical syntheses conducted. If meta-analysis was done, present for each the summary estimate and its precision (e.g. confidence/credible interval) and measures of statistical heterogeneity. If comparing groups, describe the direction of the effect. | Not applicable (qualitative synthesis). |
|  | 20c | Present results of all investigations of possible causes of heterogeneity among study results. | Not applicable (qualitative synthesis). |
|  | 20d | Present results of all sensitivity analyses conducted to assess the robustness of the synthesized results. | Not applicable (qualitative synthesis). |
| Reporting biases | 21 | Present assessments of risk of bias due to missing results (arising from reporting biases) for each synthesis assessed. | Not applicable (rapid review). |
| Certainty of evidence | 22 | Present assessments of certainty (or confidence) in the body of evidence for each outcome assessed. | Not applicable (rapid review). |
| **DISCUSSION** | | |  |
| Discussion | 23a | Provide a general interpretation of the results in the context of other evidence. | Discussed in the principal findings and recommendations for developing VHs sub-sections from Discussions. |
|  | 23b | Discuss any limitations of the evidence included in the review. | Limitations and future research sub-section from Discussion details limitations of the evidence used in the review. |
|  | 23c | Discuss any limitations of the review processes used. | Limitations and future research sub-section from Discussion details limitations of the evidence used in the review. |
|  | 23d | Discuss implications of the results for practice, policy, and future research. | Partially discussed in limitations and future research sub-section and then discussed in detail in the Conclusion. |
| **OTHER INFORMATION** | | |  |
| Registration and protocol | 24a | Provide registration information for the review, including register name and registration number, or state that the review was not registered. | Rapid Review: Expedited, not registered. |
|  | 24b | Indicate where the review protocol can be accessed, or state that a protocol was not prepared. | NA |
|  | 24c | Describe and explain any amendments to information provided at registration or in the protocol. | NA |
| Support | 25 | Describe sources of financial or non-financial support for the review, and the role of the funders or sponsors in the review. | Acknowledgments section |
| Competing interests | 26 | Declare any competing interests of review authors. | None declared as per conflicts of interest section |
| Availability of data, code and other materials | 27 | Report which of the following are publicly available and where they can be found template data collection forms; data extracted from included studies; data used for all analyses; analytic code; any other materials used in the review. | Provided as supplementary material/in appendix. |

*From:*  Page MJ, McKenzie JE, Bossuyt PM, Boutron I, Hoffmann TC, Mulrow CD, et al. The PRISMA 2020 statement: an updated guideline for reporting systematic reviews. BMJ 2021;372:n71. doi: 10.1136/bmj.n71. This work is licensed under CC BY 4.0. To view a copy of this license, visit <https://creativecommons.org/licenses/by/4.0/>
