# Supplementary material for: Advancing Regional and Remote Health Care With Virtual Hospital Implementation: Rapid Review
Source: JMIR Hum Factors. 2025 Jun 3;12:e64582. doi: 10.2196/64582 (PMC12174879; doi:10.2196/64582)
Supplement: Multimedia Appendix 2 [file humanfactors_v12i1e64582_app2.docx]

## Search Strategy

| Search String | PubMed | MEDLINE on OVID | CINAHL | Latrobe Library |
| --- | --- | --- | --- | --- |
| ("virtual hospital" OR "virtual hospital*" OR "telemedicine" OR "telehealth" OR "telemonitoring" OR "remote consultation" OR "virtual care" OR "virtual health*" OR "ehealth*" OR "mhealth*" ) AND ("regional health*" OR "rural health*" OR "remote health*" OR "remote and regional area health*" OR "rural health? services") AND ("healthcare delivery" OR "healthcare access" OR "health planning" OR "hospital strategy" OR "healthcare accessibility" OR "clinical effectiveness" OR "user experience") AND ("implementation" OR "best practices" OR "recommendations" OR "enabler" OR "strategies" OR "benefits" OR "facilitators") AND (English Language) AND (Published Date:20150101-20230301) | 61 | 33 | 60 | 168 |
| ("virtual hospital" OR "virtual hospital*" OR "telemedicine" OR "telehealth" OR "telemonitoring" OR "remote consultation" OR "virtual care" OR "virtual health*" OR "ehealth*" OR "mhealth*" ) AND ("regional health*" OR "rural health*" OR "remote health*" OR "remote and regional area health*" OR "rural health? services") AND ("healthcare delivery" OR "healthcare access" OR "health planning" OR "hospital strategy" OR "healthcare accessibility" OR "clinical effectiveness" OR "user experience") AND (challenge* OR barrier* OR problem*) AND (English Language) AND (Published Date:20150101-20230301) | 51 | 19 | 61 | 136 |
