# Supplementary material for: Advancing Regional and Remote Health Care With Virtual Hospital Implementation: Rapid Review
Source: JMIR Hum Factors. 2025 Jun 3;12:e64582. doi: 10.2196/64582 (PMC12174879; doi:10.2196/64582)
Supplement: Multimedia Appendix 3 [file humanfactors_v12i1e64582_app3.docx]

## Quality Criteria Checklist

| Study | Was the research question/objectives clearly stated? | Was the selection of study subjects/patients free from bias? | Were study groups comparable? | Was method of handling withdrawals described? | Was blinding used to prevent introduction of bias? | Were intervention/therapeutic regimens/exposure factor or procedure and any comparison(s) described in detail? Were intervening factors described? | Were outcomes clearly defined and the measurements valid and reliable? | Was the statistical analysis appropriate for the study design and type of outcome indicators? | Are conclusions supported by results with biases and limitations taken into consideration? | Is bias due to study’s funding or sponsorship unlikely? | Overall Quality Assessment |
| --- | --- | --- | --- | --- | --- | --- | --- | --- | --- | --- | --- |
| AlDossary 2017 | Yes | N/A | N/A | N/A | N/A | Yes - The framework was defined in detail | Yes - the outcome which was a framework clearly defined | N/A- no statistical analysis | Yes - the conclusion is supported by result | No Biasness due to funding | Medium |
| Davis 2020 | Yes | Yes - members were carefully selected | N/A | N/A | N/A | Yes | Yes - the outcome a multifaceted telehealth intervention was clearly defined | N/A- no statistical analysis | Yes | No Biasness due to funding | Medium |
| DeHart 2022 | Yes | Yes - providers were selected from 11 different healthcare institutions and varied in age and education levels | Yes - all providers had experience of serving rural patients | Not Specified | Not Specified | Yes , the overall methodology for conducting the research and analysing quantitative and qualitative data was described in detail | Yes, the outcomes were quantitatively described followed by qualitative evidence synthesis | Yes | Yes, conclusion was combined with discussion but it supports the results and highlights limitations | Not Specified | High |
| Giroux 2022 | Yes - the issues were highlighted clearly | N/A | N/A | N/A | N/A | Yes, the commentary provides a detail analysis on knowledge translation process for virtual care | Yes - the overarching lessons were described clearly | N/A | N/A | Not Specified | Low |
| Haque 2021 | Yes | Yes, random selection of interviewees | Yes | No | Not Specified | Yes - the exposure factors and research procedure were clearly described | Yes, outcomes were clearly discussed | N/A - No statistical findings | Yes | No | High |
| Head 2022 | Yes | No - provider judgement could have affected selection bias | Yes - all patients had at least 1 inpatient visit and 1 virtual encounter | No | Not Specified | Yes | Yes | Yes - The study presents continuous variables as mean ± SD, categorical variables as frequency and percentage, and computes the standard error of the mean (SEM) for technical issues percentage across age and sex. | Yes - limitations are defined and future directions are identified | Not Specified | Medium |
| Hirko 2020 | Yes | N/A | N/A | N/A | N/A | Yes - telehealth as intervention is described in detail | Yes - the outcomes as according to the objectives are clearly defined | N/A | Yes - The conclusion supports the discussion | Not Specified | Low |
| Howland 2021 | Yes | Yes | Yes | No | No | Yes | Yes - results were clearly analysed and described | N/A - No statistical findings | Yes - the conclusion supports results and provides limitations | Not Specified | High |
| Jong 2019 | Yes | N/A | N/A | N/A | N/A | Yes - telehealth as intervention is described properly | Yes | N/A | Yes | N/A | Low |
| LeBlanc 2020 | Yes | Yes - rigorous selection process undertaken | Yes - only retrospective studies of eHealth implementation was included | N/A | N/A | Yes - the intervention eHealth along with the methodology for conducting scoping review was comprehensively described | Yes | N/A | Yes, the conclusion supports the findings and also presents limitation | Not Specified | High |
| Thomas 2023 | Yes | Yes | Yes | No | No | Yes- detailed discussion on telehealth intervention and research method used | Yes - a detailed representation of the findings | N/A | Yes | No | High |
| Johnston 2020 | Yes | Yes | Yes | No | No | Yes | Yes - the outcomes were evaluated with evidence | N/A - No statistical findings | Yes | Not Specified | High |
| Correale 2022 | Yes | Yes- random selection of focus group members | Yes- equal distribution of allocated members | No mention of withdrawals | No - the objectives were described to the members | Yes - the RAC-LBP toolkit was described in detail | Yes- Outcomes were clearly defined | N/A- no statistical analysis | Yes - Conclusion is supported by result and also limitations are acknowledged as future research directions | No Biasness due to funding | High |
| Kocanda 2021 | Yes | Yes | Yes - all participants were rural health workers | No | No | Yes - the intervention, methodology and intervening factors were discussed | Yes | N/A - No statistical findings | Yes | No | High |
| McPherson 2021 | Yes | Yes - Random selection through social media | Yes | N/A | N/A | Yes | Yes - the results were discussed in detail | Yes - percentages used for reporting results | Yes | No | High |
| Allan 2021 | Yes | Yes - avoided potential response bias | Yes | Yes | No | Yes - The qualitative approach was described in detail | Yes- perspectives were clearly defined | N/A- no statistical analysis | Yes - the conclusion is supported by result with details to limitations | No- bias was reported due to limited participation | High |
| Nataliansyah 2022 | Yes | Yes | Yes | N/A | N/A | Yes - clear discussion on the intervention and its implementation for rural ED | Yes - clearly defined outcomes | N/A - No statistical findings | Yes - the conclusion supports the findings and also presents strengths and limitations | No | High |
| Kuperman 2018 | Yes | Yes | Yes | N/A | N/A | Yes - intervention development and Implementation was described properly | Yes- the outcomes with measurements were sound and reliable. No biasness reported | Yes | Yes - sound conclusion with discussion on limitations provided | No | High |
| Sitammagari 2021 | Yes | No - selection bias as limited to patients with telephone | Yes | No | No | Yes - the intervention along with study design was discussed properly | Yes - the outcomes were comprehensively discussed | Yes | Yes | No | Medium |
| Vindrola-Padros 2021 | Yes | Yes - a well-defined search strategy with clear inclusion and exclusion criteria | Yes | N/A | N/A | Yes | Yes | NA | Yes | No | High |
| Haleem 2021 | Yes | No - no specified inclusion or exclusion criteria | No information on how the referenced articles were cited | N/A | N/A | No | Yes - the outcomes were clearly defined and described | N/A | Yes | No | Low |
| Gray 2022 | Yes | Yes | Yes - the participation was voluntary | No | No | Yes, the entire process was clearly described | Yes | N/A - No statistical findings | Yes - the conclusion highlighted the key findings. It also addressed the pandemic as a limitation | No | High |
| Bradford 2016 | Yes | Yes - A inclusion and exclusion criteria were used | Yes - The articles selected were comparable | N/A | N/A | Yes - the systematic review methodology is described in detail | Yes - the outcomes along with findings clearly defined | N/A - No statistical findings | Yes - Conclusion is supported by result and also limitations are acknowledged as future research directions | Not Specified | High |

Table 1: Duval D, Pearce-Smith N, Palmer JC, Sarfo-Annin JK, Rudd P, Clark R. Critical appraisal in rapid systematic reviews of COVID-19 studies: implementation of the Quality Criteria Checklist (QCC). Systematic Reviews. 2023 2023/03/27;12(1):55. doi: 10.1186/

## Reference:

Duval D, Pearce-Smith N, Palmer JC, Sarfo-Annin JK, Rudd P, Clark R. Critical appraisal in rapid systematic reviews of COVID-19 studies: implementation of the Quality Criteria Checklist (QCC). Systematic Reviews. 2023 2023/03/27;12(1):55. doi: 10.1186/s13643-023-02219-z.
